# Complement C1q promotes primary haemostasis in a microfluidic bleeding model

**DOI:** 10.3389/fimmu.2026.1810209

**Published:** 2026-10-02

**Authors:** Peymane Zahiroddini, Elaissa T. Hardy, Yumiko Sakurai, Wilbur A. Lam, Marten Trendelenburg

**Affiliations:** 1 Laboratory of Clinical Immunology, Department of Biomedicine, University of Basel, Basel, Switzerland; 2 Wallace H. Coulter Department of Biomedical Engineering, Georgia Institute of Technology and Emory University, Atlanta, GA, United States; 3 Division of Internal Medicine, University Hospital Basel, Basel, Switzerland

**Keywords:** C1q, complement system, innate immunity, microfluidic bleeding model, von Willebrand factor (vWF), primary haemostasis, platelets, vascular injury

## Abstract

**Background:**

In systemic inflammatory diseases, thrombotic complications often cannot be fully explained by traditional risk factors, suggesting that the immune system directly affects hemostasis. The complement system, particularly C1q (the recognition molecule of the classical complement pathway), has been shown to enhance primary hemostasis *in vivo* and to bind von Willebrand factor (vWF) *in vitro*.

**Aim:**

To investigate the role of the C1q-vWF interaction in human primary hemostasis.

**Methods:**

We used a microfluidic bleeding model with endothelialized microchannels to visualize hemostatic processes in real-time. Following mechanical vessel injury, we tracked C1q deposition and its colocalization with vWF, platelets, and fibrinogen using confocal microscopy. The functional relevance of C1q was assessed using the C1q-inhibitory peptide 2J.

**Results:**

C1q accumulates immediately at the injury site and exhibits strong colocalization with vWF and activated platelets, while colocalization with fibrinogen is minimal. Inhibiting C1q with peptide 2J significantly reduces both C1q and vWF deposition during early clot formation, and leads to prolonged bleeding time in this model.

**Conclusion:**

Deposited C1q functions as a binding partner for vWF, enhancing primary hemostasis. This C1q-vWF axis may contribute to inflammation-related thromboembolic complications in humans.

## Introduction

Inflammation is a well-established predisposing factor for thromboembolism, with various inflammatory disorders, such as bacterial sepsis and systemic autoimmunity—frequently leading to severe thrombotic complications (1–5).

In conditions such as sepsis, activation of the immune system promotes coagulation through the generation of inflammatory mediators, including complement split products C3a and C5a, which subsequently upregulate procoagulant pathways such as tissue factor (TF) expression and activate the contact system, thereby increasing thrombogenicity. Among the components of the immune response, the interplay between the complement system and the coagulation cascade is particularly well recognized, underscoring the complex relationship between inflammation and coagulation in the pathogenesis of thromboembolic events (6–8). Both systems are considered archetypes of complex, multifunctional cascades of molecules They share evolutionary origins, structural similarities (e.g. serine proteases circulating as inactive zymogens), and functional roles as part of the body’s first line of defense against infections and injury (9, 10). While the complement system primarily offers protective functions in innate immunity against pathogens and enhances acquired immunity, its dysregulation can also be a major cause of tissue injury in various pathological conditions (11, 12).

The complement system can be activated through three distinct pathways: the classical, the lectin, and the alternative pathways (13). At the start of the classical pathway is component C1q, a large recognition protein. C1q is a multimeric glycoprotein composed of 18 polypeptide chains. These chains assemble to a distinctive hexameric structure being composed of six A, six B, and six C chains creating a structure that resembles a bouquet of tulips featuring an N-terminal collagen-like region (CLR), and six C-terminal globular head (GH) domain (14, 15). In addition to its primary function as a starter molecule of complement activation, C1q is a multifunctional protein being involved in various cellular processes, including immune cell modulation and the clearance of apoptotic cells (16, 17). Recent study suggested that C1q directly enhances primary hemostasis (3, 18–20).

The hemostatic system plays a fundamental role in maintaining vascular integrity by preventing and terminating bleeding through the coordinated actions of the vessel wall, blood cells, and the coagulation cascade (21). Hemostasis occurs via two interrelated processes: primary and secondary hemostasis. Primary hemostasis is initiated immediately after vascular injury and is characterized by the adhesion and aggregation of platelets to form a temporary platelet plug. Exposure of the subendothelial matrix activates circulating platelets and promotes their attachment to the injury site (22). A key mediator in this process is von Willebrand factor (vWF), a large multimeric glycoprotein that binds platelets to exposed collagen fibers. Structurally, vWF builds high–molecular-weight multimers that are essential for platelet adhesion under conditions of high shear stress. Shear-induced forces promote the unfolding of vWF from a compact globular conformation into an elongated structure, thereby exposing multiple functional binding sites. It also stabilizes coagulation factor VIII, linking platelet function with the coagulation cascade and thereby contributing to the secondary phase of hemostasis. Through these dual roles, vWF is central for effective hemostasis following vascular injury (23–27).

In the study presented here, we focus specifically on the functional impact of C1q on von Willebrand factor (vWF) deposition during primary hemostasis. Emerging evidence from *in vitro* models revealed a direct interaction between vWF and surface-bound C1q, underscoring a significant connection between early hemostatic events and activation of the classical complement pathway. These previous analyses demonstrated that vWF binds to immobilized C1q, particularly via its collagen-like region (CLR), promoting platelet rolling and adhesion under physiological shear stress (28–30). This interaction suggests a potential role for C1q in facilitating clot formation.

Supporting the previous *in vitro* observations, another study *in vivo* using a mouse model also suggested a functional role of C1q in primary hemostasis. C1q-deficient mice exhibited prolonged bleeding times and greater blood loss compared to wild-type controls. Notably, hemostatic function was restored upon reconstitution with C1q, highlighting its essential contribution to coagulation (31). Although a direct interaction between C1q and vWF could not be demonstrated, these findings are well in line with the view that C1q enhances primary hemostasis by promoting vWF-mediated platelet adhesion (30, 31) However, despite these recent insights, the specific interaction between complement C1q and vWF and its relevance during a hemostatic response in human blood remain to be determined in order to demonstrate that a C1q-vWF interaction occurs during primary hemostasis and enhances clot formation. For this, we utilized a microfluidic bleeding model that integrates endothelialized microchannels and a pneumatic microvalve (32). This advanced model allows real-time visualization and analysis of the hemostatic processes in whole blood following mechanical vascular injury within the artificial microvessel under flow conditions (32, 33). It enables the observation of dynamic interactions between complement C1q, platelets, von Willebrand factor (vWF), and coagulation factors, yielding results that are more compatible with human pathophysiology. The specific aims of this study were to demonstrate that complement C1q i) is deposited at the site of vessel injury, ii) is colocalizing with vWF deposition, and iii) enhances blood clot formation in this human bleeding model (32, 33).

## Materials and method

We used a microvascular bleeding model to study the role of C1q in hemostasis (32–35).

### Microfluidic bleeding device

The microfluidic bleeding devices were fabricated from polydimethylsiloxane (PDMS) in three different layers: a vascular layer (being composed of a vascular channel of 150 μm width and 50 μm height, and a side channel), a valve layer (50 μm thickness), and a valve actuator (with a valve channel of 450 um in width and 6 μm in height) (32). The vascular layer was oxygen plasma bonded using a plasma cleaner (Harrick Plasma, PDC-32G) to the valve layer, which contained a partially shielded area to prevent oxygen plasma treatment and allow the creation of the movable pneumatic valve. Inlet and outlet holes were then punched into the bonded layers and oxygen plasma bonded to the valve actuator (32–35).

Endothelialization of the microfluidic bleeding device to create an artificial microvessel

Before seeding the cells, the devices were vacuumed for 5 minutes, and all microchannels were filled with 1 mg/ml collagen type I (rat tail collagen I diluted in ddH_2_O; Thermo Fisher, Switzerland). The devices were then incubated at 37 °C for 1 hour. The side and valve channels were filled with the collagen solution, while the vascular channel was washed with ddH_2_O and filled with a fibronectin solution (0.005%; Merck diluted in DPBS). The devices were subsequently incubated for another 90 minutes at 37 °C. Then, human umbilical vein endothelial cells (HUVECs, Lonza, Switzerland) at a maximum passage of five were used. These HUVECs were cultured in CnT Endo cell culture medium (CELLnTec, Switzerland) for four days. Cells were subsequently seeded into the vascular channel at a concentration of 1 × 10^7^ cells/mL following resuspension in cell culture medium containing 8% dextran (Sigma-Aldrich) and filtration through a 35 µm cell strainer (Corning, USA). Fifteen minutes after cell adhesion, the device inlet and outlet were washed with fresh medium and further incubated at 37 °C for 2 hours before being connected to a syringe pump. The seeded cells in the device were grown under flow conditions using a syringe pump (PhD Ultra, Harvard Apparatus). The vascular channel was connected to a 10 ml syringe containing CnT Endo cell culture medium and perfused for 48 hours. The initial flow rate was set at 1 μl/min for 12 hours, followed by 2 μl/min for the remaining 36 hours, as previously described (32–35).

### Blood collection and preparation

Whole blood was obtained from anonymous healthy donors (purchased from the blood donation center in Basel, Switzerland), collected in 2.5 ml Sarstedt Monovette tubes containing 3.2% citrate (Sarstedt, Germany).

Immediately prior to use, the collected blood was mixed with 40 μg/mL corn trypsin inhibitor. (CTI; Loxo GmbH, Germany) to inhibit intrinsic pathway activation in the syringe and tubing.

### Staining of molecules

The proteins of interest were stained with fluorescently antibodies, which were added directly to the blood (32, 34). For localization experiments, fluorescently labeled or non- labeled primary antibodies followed by fluorescently labeled secondary antibodies added directly citrated whole blood (600 µl). The following primary antibodies were used: mouse monoclonal anti-C1q antibody (4 µl/600 µl blood; Bio-Rad, clone 004-43.X [3R9/2]); rabbit polyclonal anti-C1q (6 µl/600 µl blood; Dako) for experiments requiring simultaneous detection of C1q and fibrinogen, mouse polyclonal anti–von Willebrand factor (vWF) (3 µl/600 µl blood; Abcam, ab201336); rabbit polyclonal anti-vWF (3 µl/600 µl blood; Abcam, ab6994) and recombinant rabbit monoclonal anti-P-selectin (clone 3H20L10; 6 µl/600 µl blood; Invitrogen, 171257). To detect fibrinogen/fibrin, a fibrinogen–Alexa Fluor 647 conjugate was added directly to whole blood (10 µl/600 µl blood; Invitrogen, F35200; Thermo Fisher Scientific). Alexa Fluor 488–conjugated donkey anti-rabbit IgG (1.5 µl/600 µl blood; Invitrogen, A21206; Thermo Fisher Scientific) and Alexa Fluor 647–conjugated goat anti-mouse IgG (0.75 µl/600 µl blood; Invitrogen, A21236; Life Technologies) were used as secondary antibodies.

### Blood infusion to the bleeding device

The device was placed on a confocal microscope stage (Nikon Crest V3). The integrity of the cellular monolayer and the confluence of the cells within the vascular channel were confirmed by staining the HUVEC membranes with CellMask (1 μl/ml; Thermo Fisher) for 5 minutes at a flow rate of 2 μl/min. The stained whole blood was recalcified to a calcium concentration of 12.5 mM and subsequently drawn into a 1 ml syringe. Fresh, fluorescently labeled human whole blood was then infused into the endothelialized vascular channel at a shear rate of 500 s ^-1^ (flow rate 2 μl/min) (32–35).

### Induction of mechanical injury in the vascular channel

A 10 ml syringe filled with cell culture medium was connected to the side channel to induce the vessel injury, and a separate 10 ml syringe filled with distilled water (ddH_2_O) was connected to the valve channel. The valve channel syringe was installed on a withdrawal pump to create a 2 ml vacuum. Manual pressure from the side channel syringe caused an injury in the vascular channel (32–35).

### Measuring fluorescence intensity, bleeding time and quantitative analysis of C1q and von Willebrand Factor colocalization

Each experiment was conducted at least three times using blood from different donors. After mechanically injuring the microvessel, we tracked C1q at the injury site and its interactions with vWF and platelets. Confocal microscopy enabled detailed imaging and quantification of the fluorescence signal intensities of C1q and other proteins of interest, providing insight into the contribution of C1q to the hemostatic process in real-time. The analyses performed by confocal microscopy involved several key steps to determine the deposition and colocalization of components of interest at the injury site in the microfluidic bleeding model. Initially, the model was established with a confluent monolayer of human endothelial cells. Upon mechanical injury, the hemostatic response was observed using a confocal microscope (Nikon Crest V3 – Cresty) for a maximum of 30 minutes, during which images were recorded at one-minute intervals. The size of the injury was measured in the first frame using Fiji software (Fiji - doi: http://doi.org/10.1038/nmeth.2019). A region of interest (ROI) measuring 450 x 300 μm² surrounding the injury site was selected to evaluate changes in fluorescence intensity. The Time Series Analyser plugin of Fiji tracked the signal intensity of the region of interest (ROI) over time, enabling the calculation of fluorescence increases compared to the initial time point. Bleeding time was visually assessed, defined as the moment when no blood flow was observed any more toward the side channel. Fluorescently labeled antibodies were employed to visualize specific proteins, including C1q and vWF, as outlined before. The images captured at various time points allowed for the assessment of colocalization between these components.

Colocalization between C1q and von Willebrand factor (vWF) was quantified at each time point using Fiji software (Fiji - doi: http://doi.org/10.1038/nmeth.2019). Identical imaging settings and analysis parameters were maintained across all time points to ensure comparability between samples. Fluorescence channels for C1q and vWF were processed independently. Threshold values were determined empirically from representative images and kept constant across all analyses, with automatic threshold recalculation disabled to preserve consistency. Binary masks were generated for both channels using these fixed thresholds. Overlapping regions between C1q and vWF positive areas were identified with the logical AND operation between the two binary masks. For each time point, this workflow produced three binary images: (i) the C1q-positive mask, (ii) the vWF-positive mask, and (iii) the colocalized C1q/vWF mask. Positive areas for each mask were measured and reported in square micrometers (µm²). Colocalization was expressed as the percentage of overlapping area relative to the total positive area of each individual channel, calculated as:


$$\text{Colocalization }(\%)=(\frac{\text{Area of C}1\text{q‐vWF overlap}}{\text{Area of C}1\text{q‐positive or vWF‐positive mask}})\times 100$$

### Impact of complement C1q on vWF binding and clot formation

To investigate the functional relevance of C1q interactions in hemostasis, we assessed bleeding time and the fluorescence intensities of deposited vWF and CD62P in whole blood supplemented with C1q-inhibitory versus control peptides. The inhibitory peptide 2J (20 µg/ml) was used to block C1q function. Peptide 2J (CEGPFGPRHDLTFC) contains two cysteine residues enabling formation of an intramolecular disulfide bond, generating a cyclic structure required for high-affinity binding to the globular head domain of C1q and subsequent inhibition of its interaction with target molecules such as IgG (36). As controls, we included the irrelevant C08 peptide (20 µg/ml), previously described as a non-inhibitory sequence derived from the N-terminal region of the C1q-C chain (GAPGKDGYDGLPG) (37). All peptides were added directly to freshly collected whole blood prior to assay initiation.

## Results

To study the role of complement C1q, the initiating component of the classical pathway, in hemostasis, we employed a microvascular bleeding model. Real-time confocal microscopy was used to visualize the process by specifically staining C1q and the hemostatic proteins and cells of interest.

### C1q accumulation at the injury site and colocalization with von Willebrand Factor

Following mechanical injury to the vessel, blood flow occurred through both the vascular and wound channels. In the first set of experiments the C1q signal was already apparent in the first image of the time series, acquired 1-minute post-injury (**Figure 1**). C1q localized at the vessel edge, directly at the injury site, and on injured cells. Over time, the signal intensified and formed a wall-like structure at the site where blood exited the vessel, reaching a plateau in staining intensity after about 10 min (**Figure 2B**; **Supplementary Figure 1**). We also visualized vWF at the injury site (**Figure 2A**). As with C1q, the vWF signal was evident in the first image of the time series, acquired 1 min after injury. However, in contrast to C1q, vWF staining intensity continued to increase throughout the experiment. Notably, both C1q and vWF staining were clearly restricted to the injury site and exhibited strong colocalization starting from the earliest imaging time point, supporting the hypothesis that C1q interacts with vWF to promote hemostatic plug formation (**Figure 2B**; **Supplementary Figure 1**). Control experiments produced no fluorescence signal above background, confirming the specificity of the observed C1q staining (**Supplementary Figure 4**). We next investigated temporal changes in C1q–vWF colocalization by quantitatively analyzing their overlap at multiple time points between 5- and 30-min post-injury. The proportion of C1q that colocalized with vWF was highest at 5 min after injury and decreased at later time points (**Supplementary Figures 2A, B**).

**Figure 1:**
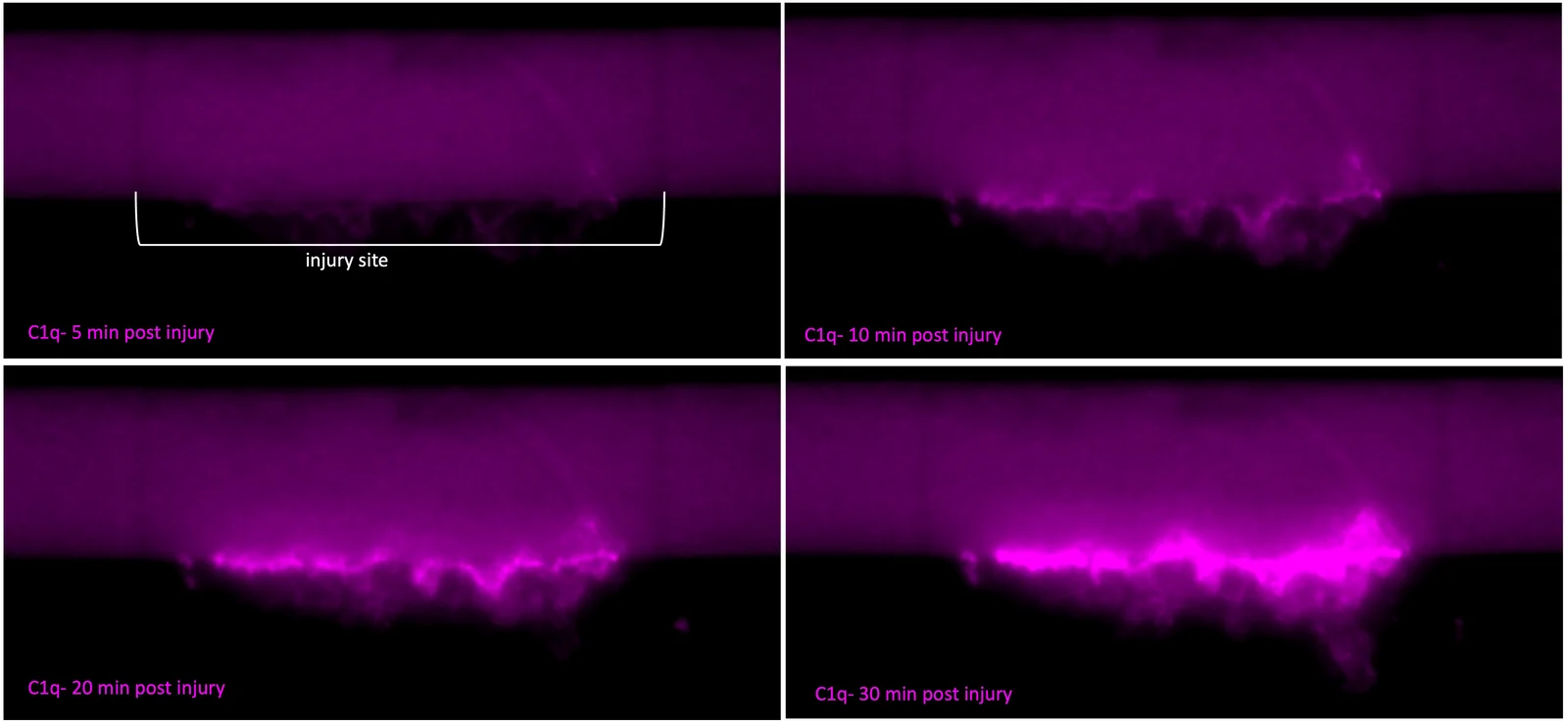
C1q accumulation at the injury site. Time-series images showing the accumulation of stained C1q (magenta) as a wall-like structure at the injury site in the microfluidic bleeding model, shown from top left to bottom right: 5, 10, 20, and 30 minutes post-injury. Scale bar, 50 μm. C1q signal intensity increased over time (**Supplementary Figure 1**).

**Figure 2:**
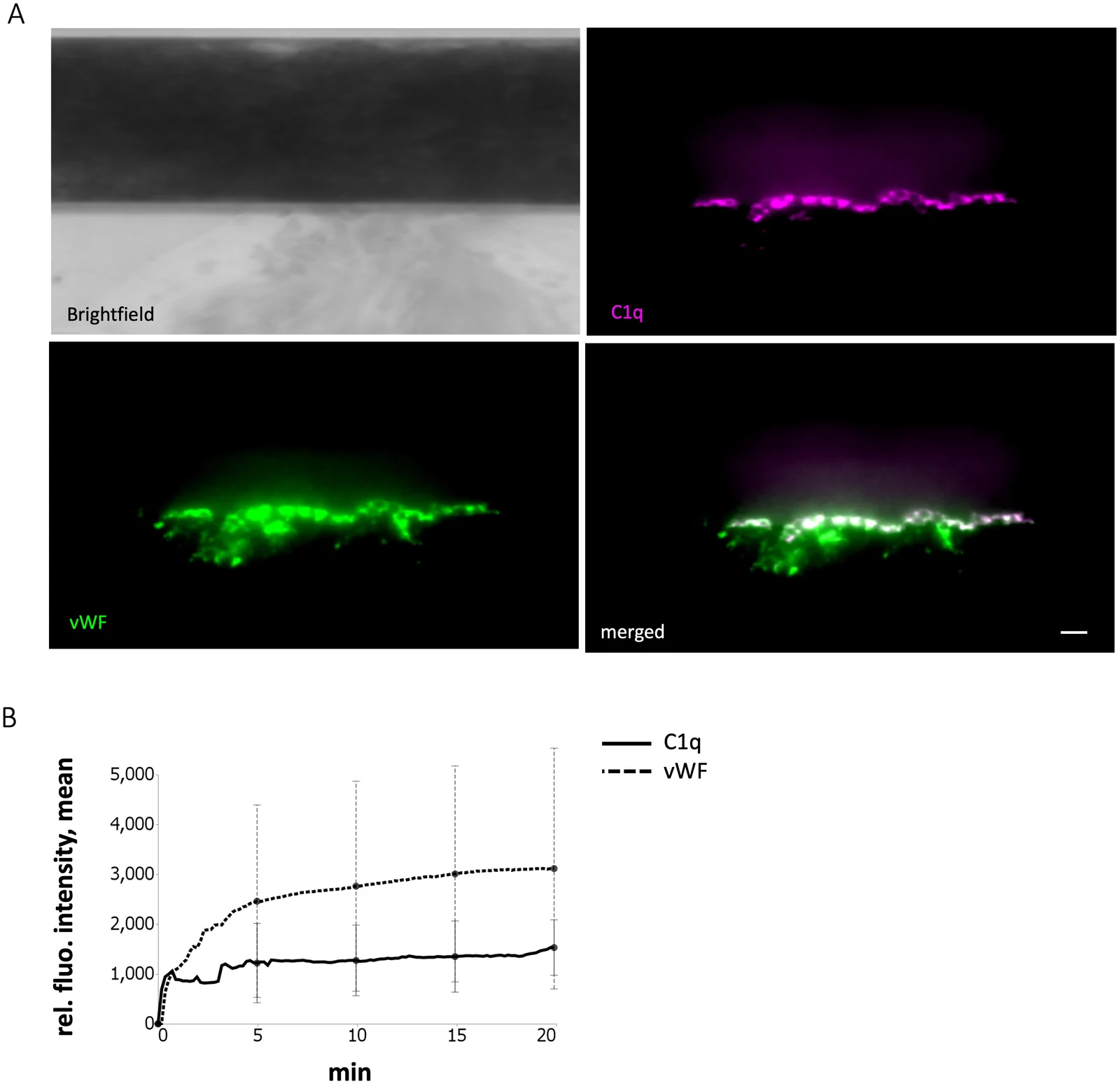
C1q-vWF colocalization upon vessel injury. **(A)** Time series images from the microfluidic bleeding model, shown from top left to bottom right: brightfield, deposition of C1q (magenta), vWF (green), and merged signals (white) at the injury site 30 minutes post-injury. Scale bar, 50 μm. **(B)** Quantitative analysis showing that both signals were detectable already 1-minute post-injury, with intensities increasing over time. The error bars indicate standard deviation from three independent replicates.

### C1q colocalizes with platelets at the injury sites but only minimally with fibrinogen

Next, we aimed to explore other components of the coagulation system which might interact with deposited C1q. For this, we first investigated a potential interaction between C1q and activated platelets being stained by an anti-CD62P (P-selectin) antibody. Again, both C1q and platelet signals were observed already in the first image of the time series (1 min after injury) at the injury site over time. Additionally, C1q showed strong colocalization with CD62P positive platelets resembling the data as observed for C1q and vWF, forming a distinct wall-like structure at the vessel outflow region that indicated substantial overlap of their signals at the injury site (**Figure 3**). In contrast, C1q showed only minimal colocalization with fibrinogen (**Figure 4**), indicating that its association with fibrinogen is limited or even absent. The weak colocalization is consistent with the broader distribution of fibrinogen throughout the vessel and collagen pouch, compared to the more restricted localization of C1q at the injury site.

**Figure 3:**
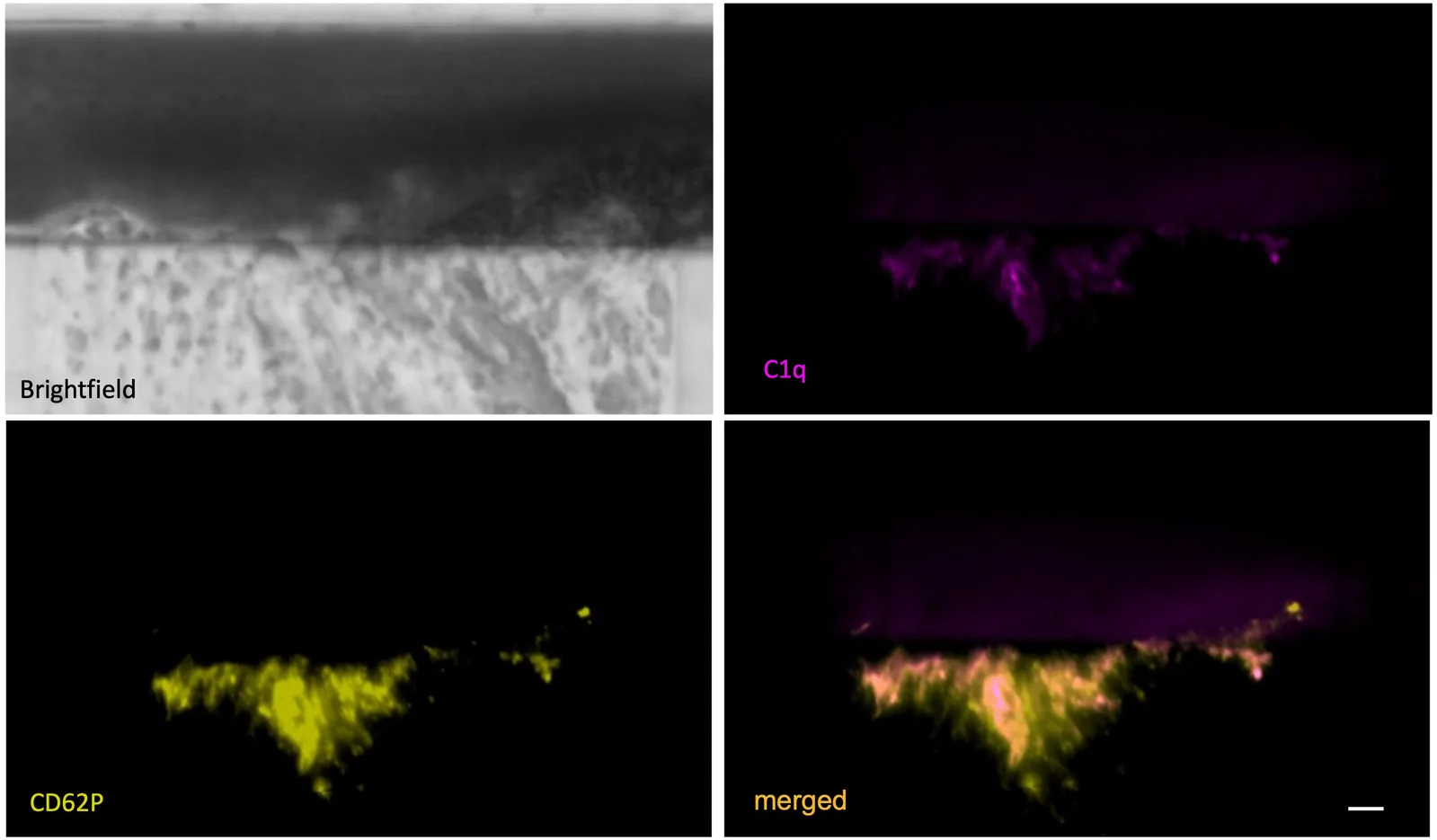
C1q colocalizes with activated platelets at the site of vessel injury. Time-lapse images (top left to bottom right) showing a brightfield view, C1q staining (magenta), CD62P (P-selectin, yellow), and merged signals (orange) at the injury site 30 minutes post-injury. CD62P and C1q were fully colocalized. Scale bar: 50 μm.

**Figure 4:**
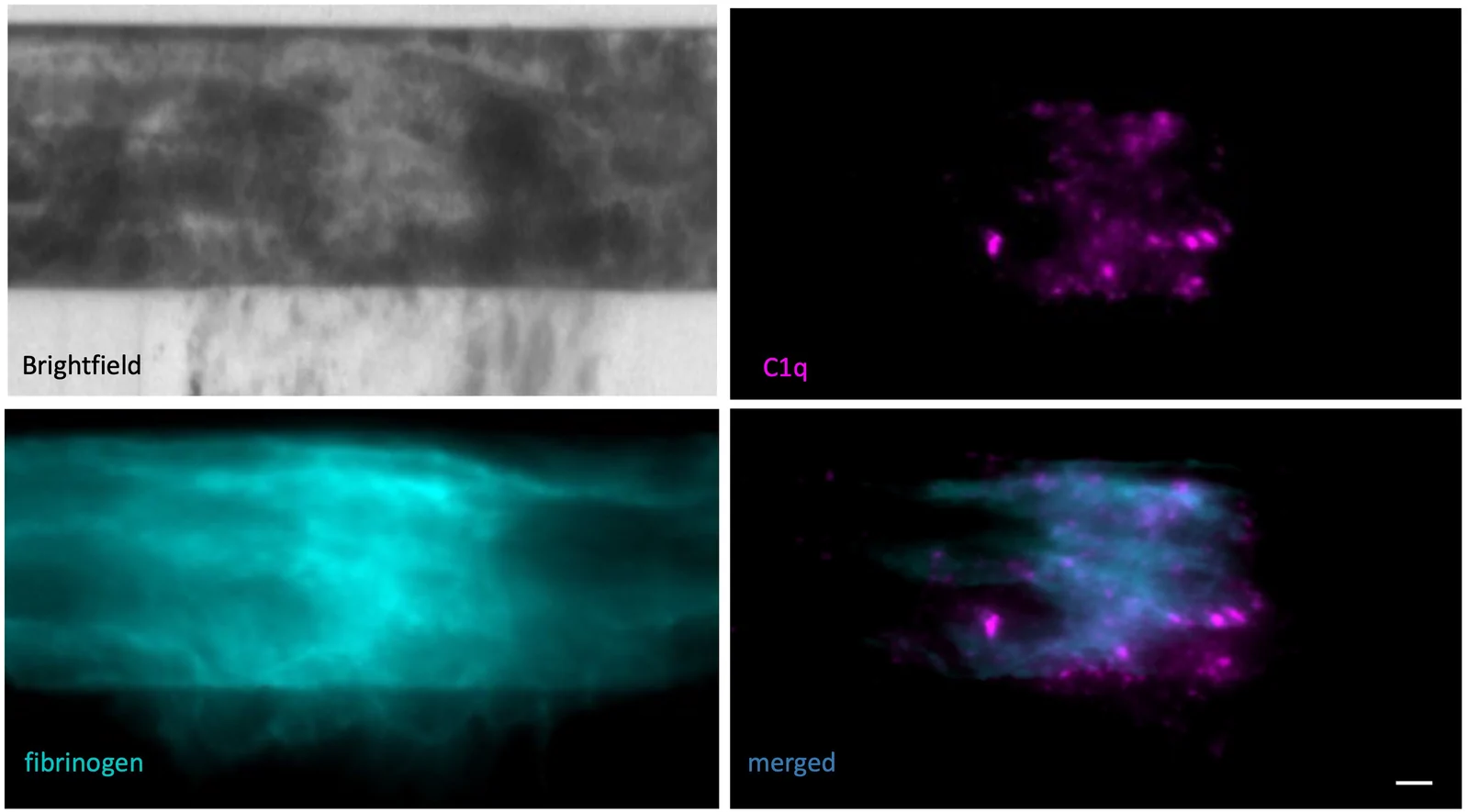
Partial colocalization of C1q and fibrinogen at the site of vessel injury. Time-lapse sequence (top left to bottom right) showing brightfield view, C1q staining (magenta), fibrinogen-AlexaFluor™647 conjugate (cyan), and merged channels (violet) at the injury site 30 minutes post-injury. C1q exhibits partial colocalization with fibrinogen. Scale bar: 50 μm.

### Immediate and parallel recruitment of C1q and vWF to the injury site

To dissect the temporal sequence of the deposition of C1q and von Willebrand factor (vWF) at the injury site, we then performed high-frequency imaging, acquiring images at 10-second intervals post-injury. Again, signals for deposited C1q and vWF became visible very early on, i.e. within less than a minute. Of note, C1q appeared to become detectable slightly earlier than vWF at the injury site (already within the first 10 seconds) (**Figure 5**). These results indicate that both, C1q and vWF represent the first line of reaction and that their interaction may immediately follow vascular injury.

**Figure 5:**
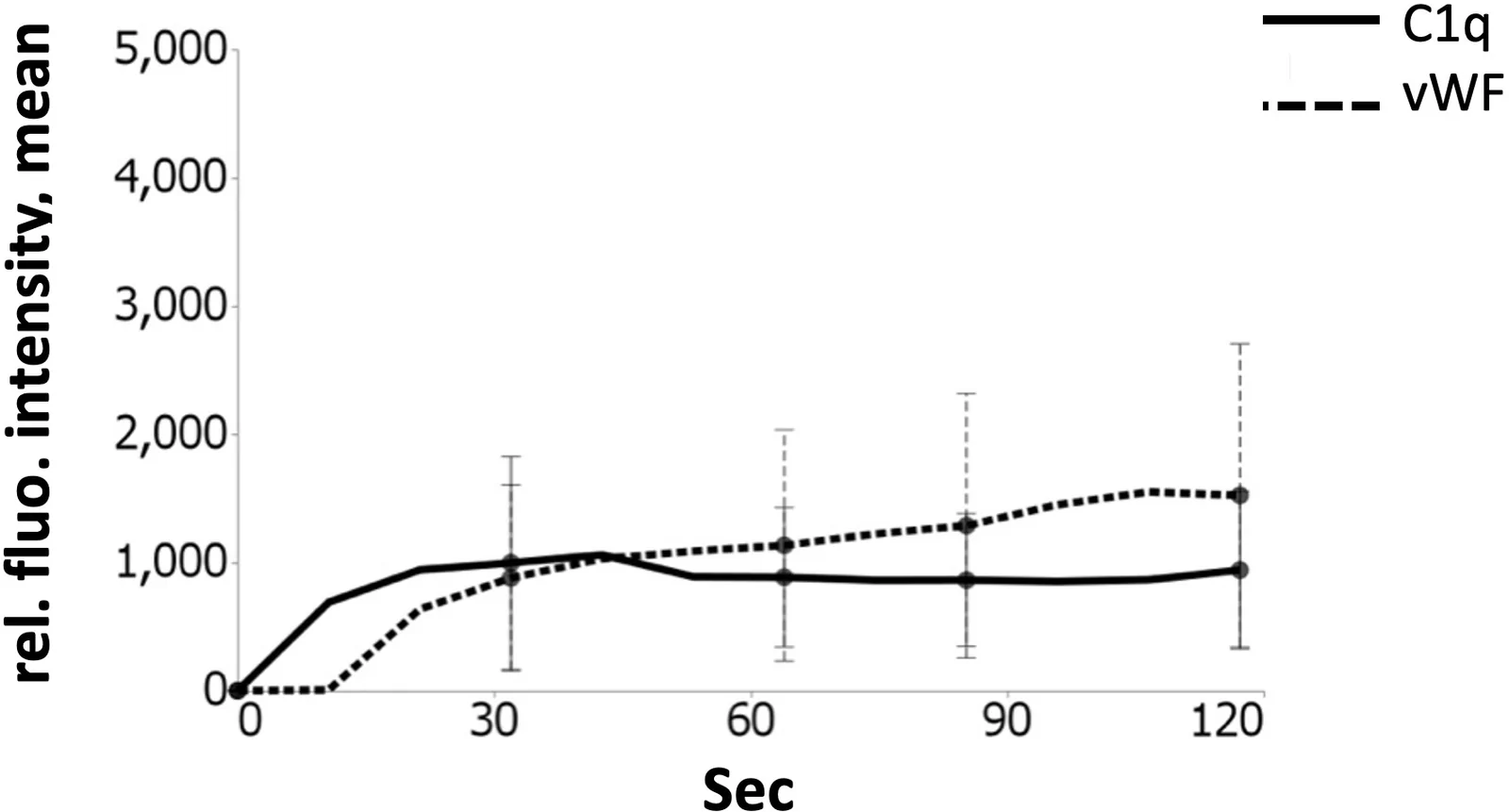
Parallel deposition of C1q and vWF following vascular injury as investigated by high-frequency imaging. Simultaneous and parallel recruitment of C1q and von Willebrand factor (vWF) at the injury site, imaged at 10-second intervals.

### Temporal dynamics of C1q–vWF colocalization at the injury site

To characterize the temporal dynamics of C1q and von Willebrand factor (vWF) colocalization following mechanical injury, we performed quantitative analysis of their spatial overlap at multiple time points between 5- and 30-min post-injury (**Figure 6A**). This analysis revealed significant time-dependent variations in colocalization patterns (**Figures 6A, B**). Specifically, the proportion of C1q colocalized with vWF peaked at 5 min and then steadily declined at later time points. In contrast, the percentage of vWF associated with C1q was lowest at early time points but progressively increased over the 30-min observation period (**Figure 6B**).

**Figure 6:**
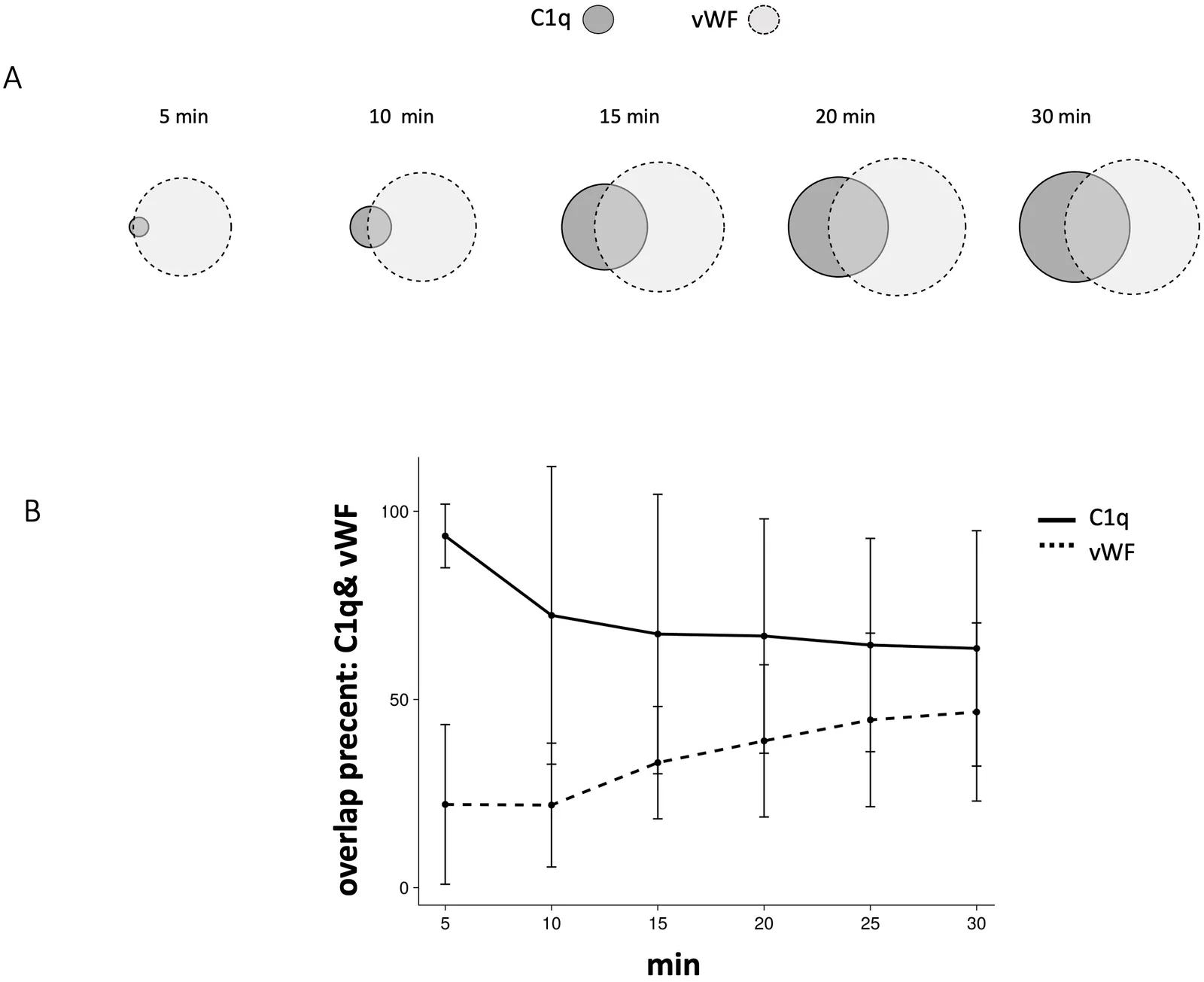
Quantitative analysis reveals time-dependent changes in C1q and von Willebrand factor colocalization. **(A)** Proportional Venn diagrams (C1q: dark gray; vWF: light gray) averaged from three independent experiments, representing measured surface areas (µm²) and their pairwise overlap at approximately 5, 10, 15, 20, and 30 minutes. Circle sizes are scaled such that their areas are proportional to the measured area for each species, and the inter-center distance is chosen so that the geometric overlap matches the measured overlap area. Thus, these plots visually convey both absolute coverage and the degree of colocalization between C1q and vWF over time. **(B)** Comparison of colocalization percentages between C1q and vWF over a 30-minute post-injury period.

### Inhibiting C1q binding reduces deposition of von Willebrand Factor and prolongs bleeding time after vascular injury

Last, we aimed at investigating the effects of inhibiting C1q on vWF deposition, and bleeding time comparing two different peptides: the inhibitory cyclic peptide 2J (2J) and the non-inhibitory C1q-derived peptide C08 (36, 37). The C1q inhibitory peptide 2J binds to the globular head (GH) domains of C1q, preventing interactions with target structures (**Figure 7**) (36). In contrast, peptide C08 has no inhibitory effects on C1q (**Figure 7**). As expected, treatment with peptide 2J markedly reduced C1q binding at the injury site. Notably, vWF deposition at the injury site was also reduced, accompanied by a prolonged bleeding time in the presence of 2J (bleeding time of 30min on average in the presence of 2J versus 20min on average for the control peptide). Of note, while the comparison of three separate sets of experiments, each revealing highly variable kinetics, did only partially allow to generate significant differences on averaged data, decreased deposition of C1q and vWF after the use of peptide 2J was observed in every single experiment (at least three repetitions).

**Figure 7:**
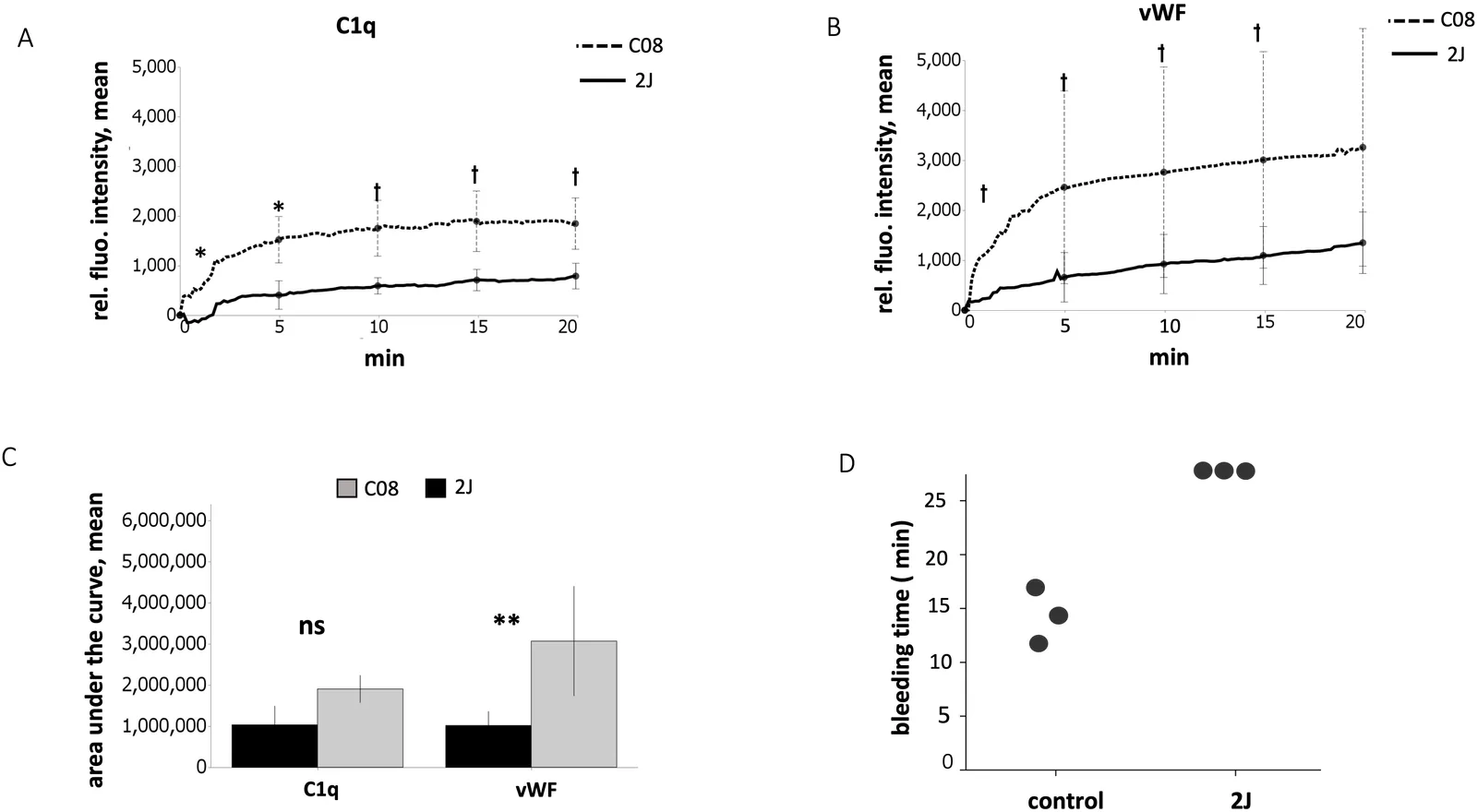
Inhibition of C1q binding reduces the accumulation of C1q as well as the deposition of von Willebrand factor at the injury site, and increased the bleeding time. **(A)** Quantitative comparisons of normalized signal intensities for C1q, **(B)** and von Willebrand factor (vWF) in the presence of the inhibitory peptide 2J, compared to the non-inhibitory control peptide C08. Statistical differences are based on paired t-tests comparing each timepoint. Significance levels: a single asterisk (*) indicates a significant difference with p< 0.05. A dagger (†) suggests a trend toward significance with p-values between 0.05 and 0.1. **(C)** Comparison of signal intensities of C1q and vWF in the presence of the C1q inhibitory peptide 2J versus the control peptide C08 across all time points by calculating the area under the curve. Significance was determined by unpaired t-tests (*p ≤ 0.05, **p ≤ 0.01, ns, not significant). Data represent mean ± SD from three independent experiments. **(D)** Treatment with peptide 2J prolonged bleeding time compared to the control peptide (maximum experimental duration: 30 min; n = 3).

In addition, treatment with 2J numerically also reduced platelet activation, as indicated by a decreased CD62P signal (**Supplementary Data 3**).

## Discussion

Increasing evidence indicates a substantial crosstalk between the complement and coagulation systems (38–41). Elucidating this complex interplay is critical for defining the mechanisms underlying thrombotic complications in inflammatory conditions, such as infectious diseases, sepsis and autoimmune diseases (42–44). Previous studies suggested that C1q, a key component of the classical complement pathway, may support hemostasis by interacting with vWF and platelets and thus may emerge as a potential target in the context of thrombotic disease (30, 31). However, the role and physiological relevance of these interactions remain incompletely understood, particularly in experimental systems that closely reflect the human *in vivo* situation.

Therefore, in the present study, we investigated the interaction between C1q and vWF and assessed the functional impact of C1q on hemostasis using a microfluidic bleeding model that mimics physiological flow conditions. This approach enabled direct, real-time visualization of C1q during blood clot formation and provided mechanistic insights into its role in early hemostatic processes.

We showed that following vascular injury, C1q rapidly (i.e. within seconds) accumulates at the site of injury, particularly enriched in areas of exposed endothelium and collagen, forming a distinct, wall-like structure. The observation of C1q accumulation directly at the injury site is a novel and intriguing finding, and suggests a role for C1q in hemostasis. In line with this hypothesis, we could detect vWF at the site of the injury showing clear colocalization with C1q within seconds after the vessel injury. This observation is well in line with our previous studies showing that vWF can bind very efficiently to surface-bound C1q under shear stress and that this interaction involves the N-terminal arginine-rich domain of the C1q A chain of the collagen-like region of C1q (30). In this context it may also be of interest to note, that the initial identification of a C1q-vWF interaction was based on the observation of a sequence homology between vWF and monoclonal anti-C1q autoantibodies (30). Nevertheless, while the close spatial and temporal congruence of C1q and vWF observed in the present human whole-blood model, together with our previous experimental findings, supports the view that these molecules directly interact early after vascular damage, we cannot exclude an additional role for bridging molecules that may enhance the described C1q–vWF interaction when using whole blood.

The initial appearance of C1q slightly prior to the first vWF deposition suggests a role for C1q in the earliest steps of hemostasis and supports the view that C1q may act as a binding partner for vWF with a direct interaction between vWF and C1q *in vivo*. In addition, our quantitative analysis of C1q-vWF colocalization over multiple time points revealed a decrease in overlap after 5 minutes suggesting that C1q and vWF play a critical functional role during the early phase of the hemostatic response, whereas their relative interaction and contribution seem to diminish at later stages.

We also demonstrate that C1q colocalizes with activated platelets at the injury site, consistent with prior reports describing dynamic interactions between the complement system and platelet activation (28, 29, 45). Taken together, our observations suggest that C1q may indirectly or directly contribute to platelet activation and deposition, and thus forms a close relationship between C1q, vWF and platelets.

In contrast, our data also suggest that fibrinogen is less likely to be the primary binding partner for C1q. This finding is consistent with previous studies showing that C1q does not directly bind to fibrinogen but instead binds to fibrin, the polymerized form of fibrinogen (38, 46). Collectively, these results highlight the distinct contributions of C1q to hemostasis, particularly its preferential interaction with vWF and platelets, and reveal a more complex role for C1q in hemostasis than previously appreciated.

Finally, we evaluated the functional relevance of C1q in the hemostatic response following vascular injury. An inhibitory peptide 2J was used to block the globular heads of C1q (36). As expected, C1q incorporation into the clot was reduced. Of interest, inhibition of C1q deposition also markedly decreased vWF deposition as well as platelet activation and accumulation at the injury site, further supporting a direct interaction between C1q, vWF, and platelets. Importantly, this event was associated with a prolongation of bleeding time, underscoring the critical role of C1q in promoting hemostasis.

Taken together, our findings provide the first evidence, using a human *in vitro* model, that complement C1q, the starter molecule of the classical pathway, may have evolved as a complementary system that supports hemostasis. Our results demonstrate that C1q interacts with vWF at sites of vascular injury and promotes early hemostatic responses which in turn shorten the bleeding time in a microvascular human bleeding model. These observations provide new insights into complement–hemostasis cross-regulation by revealing the C1q-vWF axis, which seems to be beneficial for physiological hemostasis but may drive thrombus formation under inflammatory conditions.

A key strength of our microfluidic bleeding model is its ability to visualize, in real time, the interactions between C1q, von Willebrand factor (vWF), and platelets in an endothelialized artificial vessel perfused with human whole blood under physiological shear conditions. This setup allows precise analysis of hemostatic dynamics in a microenvironment that mimics *in vivo* vascular injury. Our study is nevertheless based on an *in vitro* model and therefore may still not fully reflect human physiology. However, the chosen model uses human whole blood and mimics the vascular environment as closely as currently possible in an experimental setting. In addition, our findings are consistent with previous studies investigating not only the C1q–vWF interaction but also its potential physiological consequences (30, 31).

Our study has several additional limitations. Due to practical constraints of the highly complex model including the use of freshly drawn whole blood from anonymous healthy donors and due to limitations in visualizing several different molecules at the same time due to interactions between the staining antibodies, we focused exclusively on the interaction between C1q and von Willebrand factor (vWF) within the hemostatic system, and restricted both the number of experimental replicates as well as the range of conditions tested. In addition, we did not investigate tissue factor or other coagulation factors that might interact with C1q as well. Regarding C1q staining we used anti-C1q antibodies and cannot fully exclude that these antibodies affected C1q binding and/or function. To verify our data, we used two different anti-C1q preparations for visualization and, as shown in our experiments, C1q deposition did not appear to be altered by the presence of the used antibodies. However, the anti-C1q antibodies used for staining still might have affected downstream complement activation. Last, including blood from C1q-deficient patients would have provided further insights, but unfortunately was not accessible here in satisfying amounts and considering the time-critical set up within the experimental procedure.

In summary, our findings support the hypothesis that C1q substantially contributes to the hemostatic response by promoting vWF accumulation and platelet activation, thereby reducing bleeding time. The results indicate that C1q is not only involved in activation of the classical complement pathway but may also reinforce primary hemostasis. Although this complement C1q - coagulation crosstalk can be expected to be beneficial in the context of mechanical vessel injury, we speculate that it may also contribute to pathological thrombus formation in inflammatory vascular disease states.

## Data Availability

The raw data supporting the conclusions of this article will be made available by the authors, without undue reservation.
