# Supplementary material for: Complement C1q promotes primary haemostasis in a microfluidic bleeding model
Source: Front Immunol. 2026 Oct 2;17:1810209. doi: 10.3389/fimmu.2026.1810209 (PMC13671985; doi:10.3389/fimmu.2026.1810209)
Supplement: Supplementary file 1 [file DataSheet1.pdf]

Supplementary Materials

Supplementary Figure 1

https://app.koofr.net/links/2083299f-4018-4551-9fce-56231deeb5a3
The videos show, that the accumulation of both, C1q and vWF at the injury site increases over time, along with colocalization of C1q and von Willebrand factor (vWF). The video was produced from images recorded every minute over a 45-min period (2700 s total).

Supplementary Figure 2

A

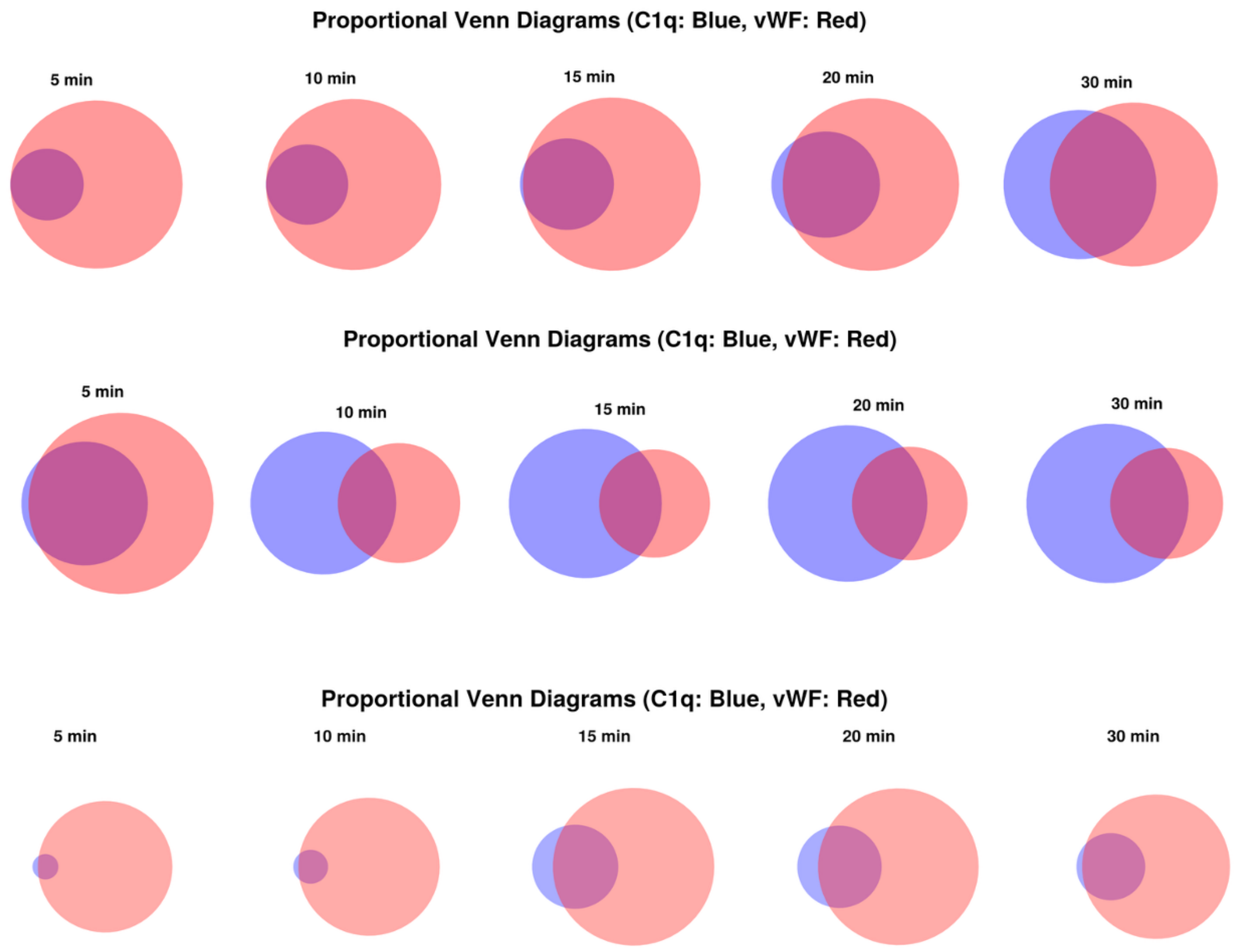

B

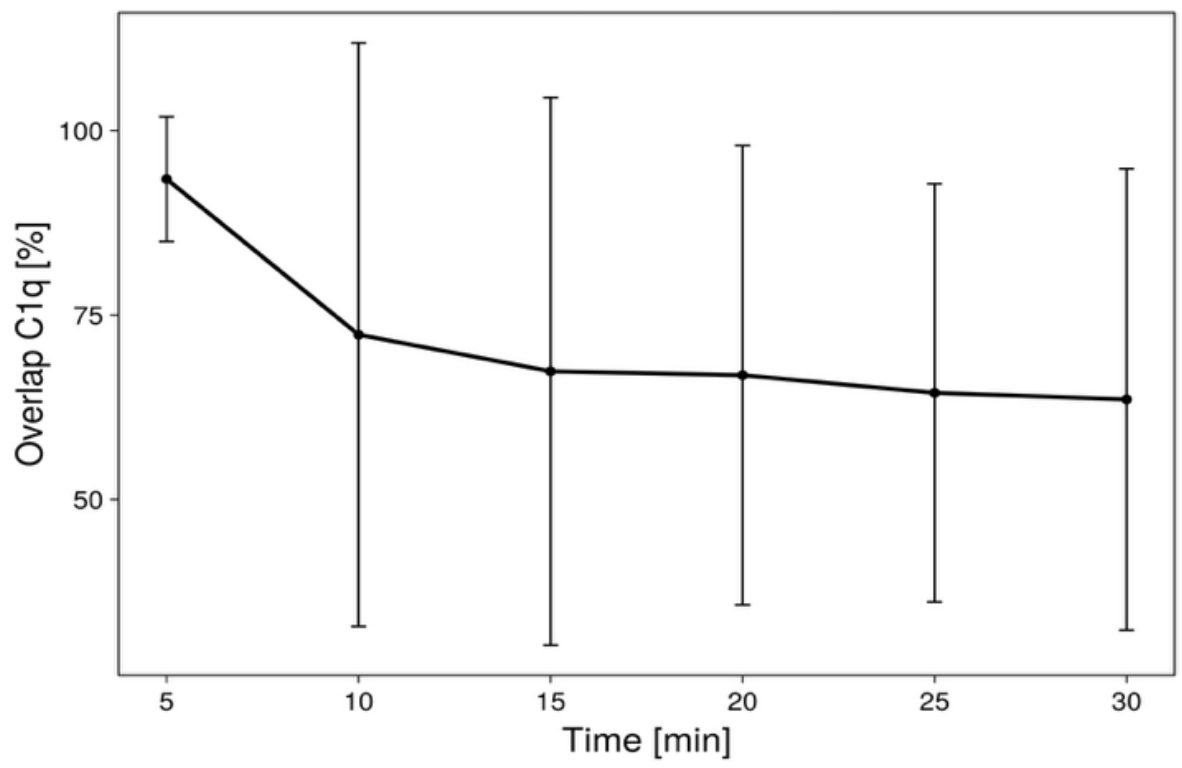

Quantitative analysis reveals time-dependent changes in C1q and von Willebrand factor colocalization

(A) Proportional Venn diagrams (blue: C1q; red: vWF) from three independent experiments, representing measured surface areas (µm²) and their pairwise overlap at approximately 5, 10, 15, 20, and 30 minutes (corresponding to the closest measured time point for each target). Circle sizes are scaled such that their areas are proportional to the measured area for each species, and the inter-center distance is chosen such that the geometric overlap matches the measured overlap area. Accordingly, the plots visually convey both absolute coverage and the degree of co-localization between C1q and vWF over time. (B)Time dependent colocalization of C1q is shown across the 5-30 min post injury time course. The results show time dependent variation in the degree of colocalization.

Supplementary Figure 3

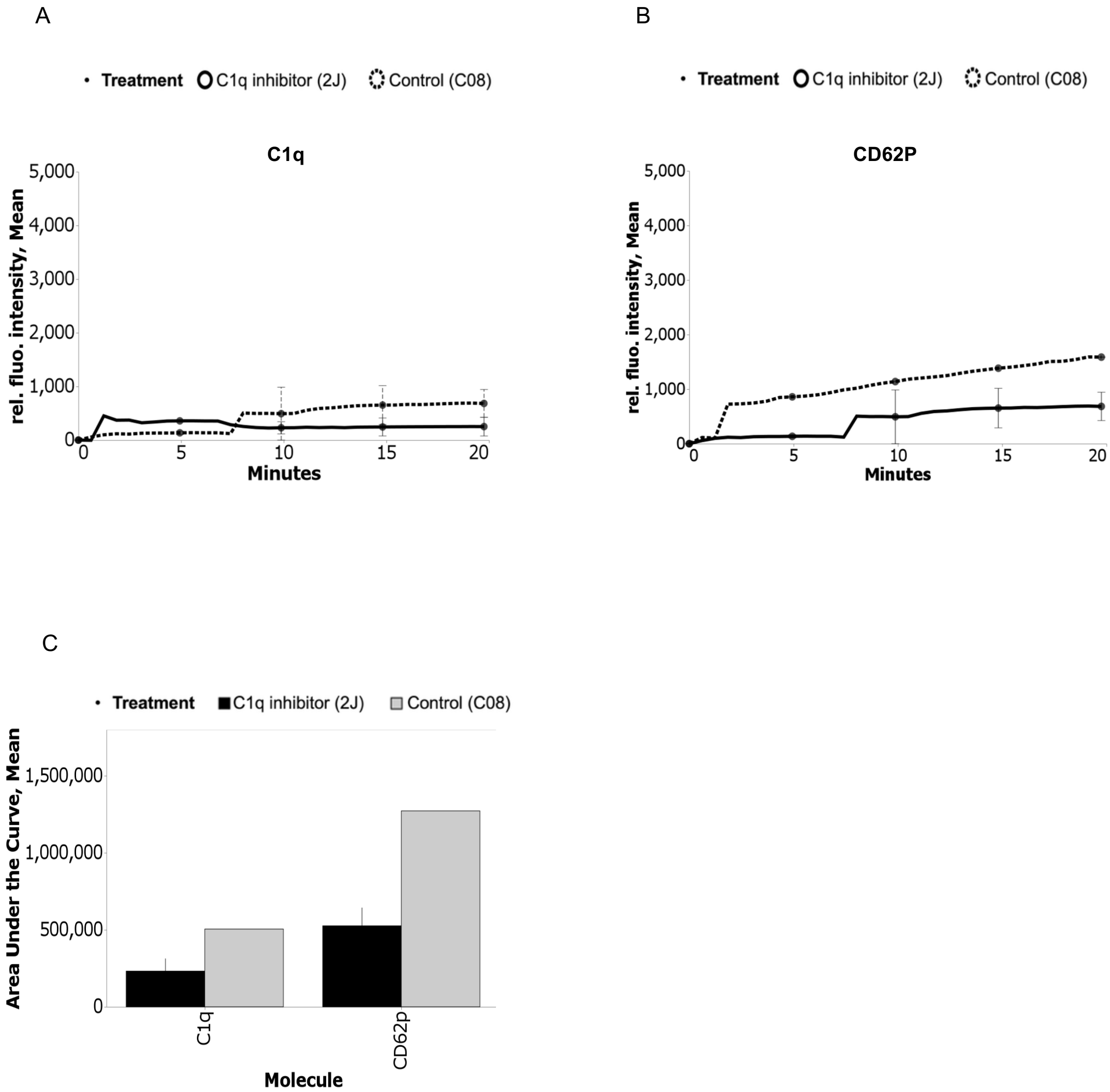

Supplementary Figure 3. Inhibition of C1q binding reduces platelet accumulation at the injury site

(A) Time-dependent changes in normalized signal intensity for C1q and (B) platelets in the presence of the inhibitory peptide 2J, compared to the non-inhibitory control peptide C08. (C) Comparison of signal intensities of C1q and platelets in the presence of the C1q inhibitory

peptide 2J versus the control peptide C08 all time points by calculating the area under the curve. No statistical test for detection of significant differences could be performed as we had only one replicate per control.

Supplementary Figure 4

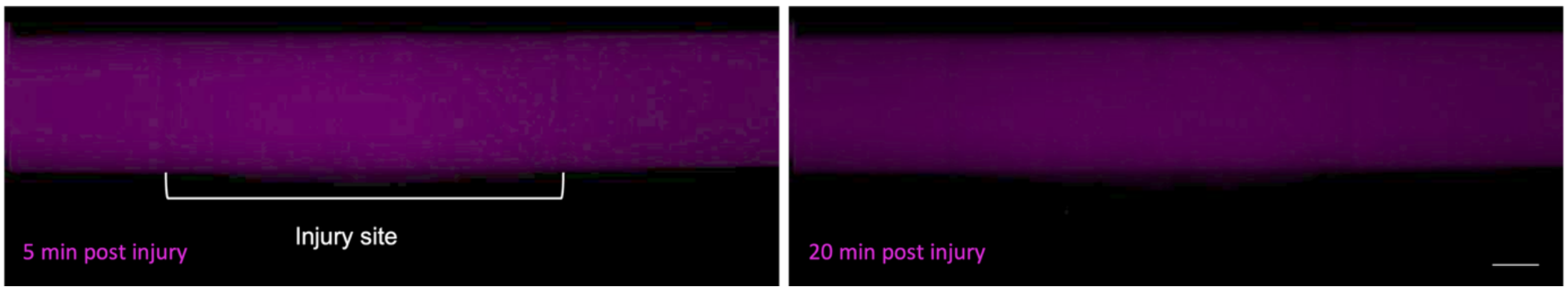

Supplementary Figure 4 | Isotype control validates the specificity of anti-C1q immunostaining in whole blood.

Whole blood was stained with a mouse IgG1 isotype control antibody (4 µl/600 µl blood; Cell Signaling Technology, 5415S) at the same concentration as the anti-C1q primary antibody, followed by the same Alexa Fluor 647–conjugated goat anti-mouse IgG secondary antibody (0.75 µl/600 µl blood; Invitrogen, A21236). Representative fluorescence micrographs show no signal above background in the device, confirming that the signal observed with the anti-C1q antibody is not due to non-specific binding of the secondary antibody, non-specific adsorption of mouse IgG to the vessel surface, or non-specific deposition of immune complexes. Scale bar: 50 µm.
